# NITPICK: peak identification for mass spectrometry data

**DOI:** 10.1186/1471-2105-9-355

**Published:** 2008-08-28

**Authors:** Bernhard Y Renard, Marc Kirchner, Hanno Steen, Judith AJ Steen, Fred A Hamprecht

**Affiliations:** 1 Interdisciplinary Center for Scientific Computing, University of Heidelberg, Heidelberg, Germany; 2 Department of Pathology, Children’s Hospital Boston, Boston, MA, USA; 3 Department of Pathology, Harvard Medical School and Children’s Hospital Boston, Boston, MA, USA; 4 Department of Neurobiology, Harvard Medical School and Department of Neurology, Children’s Hospital Boston, Boston, MA, USA

## Abstract

**Background:**

The reliable extraction of features from mass spectra is a fundamental step in the automated analysis of proteomic mass spectrometry (MS) experiments.

**Results:**

This contribution proposes a sparse template regression approach to peak picking called NITPICK. NITPICK is a Non-greedy, Iterative Template-based peak PICKer that deconvolves complex overlapping isotope distributions in multicomponent mass spectra. NITPICK is based on *fractional averagine*, a novel extension to Senko's well-known averagine model, and on a modified version of sparse, non-negative least angle regression, for which a suitable, statistically motivated early stopping criterion has been derived. The strength of NITPICK is the deconvolution of overlapping mixture mass spectra.

**Conclusion:**

Extensive comparative evaluation has been carried out and results are provided for simulated and real-world data sets. NITPICK outperforms pepex, to date the only alternate, publicly available, non-greedy feature extraction routine. NITPICK is available as software package for the R programming language and can be downloaded from .

## Background

The reliable extraction of proteomic features from complex biological mixtures is of utmost interest for unraveling the intricate biomolecular interplay at the heart of many systems biology research questions. In this context, mass spectrometry (MS) has become a key technology which provides peptide and protein identification, modification characterization and quantification capabilities. In contrast to gene expression microarray technologies, MS analysis yields a direct view on the whole set of proteins (the proteome) present in the system under investigation and can thus contribute to a richer picture of protein interaction, real-time dynamics and their regulation [1]. MS contributes to clinical research and the diagnosis process [2], it is used to detect, grade and characterize cancer diseases [3], it serves as a general purpose tool for microorganism characterization [4,5] and provides sensitive and specific means for pharmaceutical quality control.

MS experiments typically contain tens to thousands of spectra, each of which holds intensity information for tens to hundreds of thousands of mass channels. These data stem from a set of different mass analysis technologies, combining chemical separation procedures (chromatography), ionization methods (electrospray ionization, matrix-assisted laser desorption/ionization) and mass analyzers (time-of-flight, quadrupole, ion cyclotron motion). Despite physicochemical preprocessing and the availability of high mass resolution instruments, spectra which stem from complex biochemical mixtures (e.g. cell lysate, blood or serum) frequently exhibit overlapping isotope distributions of independent molecular species. Moreover, in many quantitative MS approaches, these mixtures are present by design and their manual unmixing, quantification and interpretation is tedious or unfeasible.

As a consequence, the automated analysis and interpretation of multicomponent mass spectra is highly desirable. An (incomplete) set of challenges for MS feature extraction includes the sheer data set sizes, mixtures of isotope patterns, the presence of multiple charge states, chemical and detector noise, species-dependent ionization efficiencies, chemical reproducibility and deviations from detector linearity. Among all requirements that derive from these challenges, it is important to emphasize the crucial nature of the feature extraction step: as all subsequent analysis steps rely on the set of extracted features, meaningful biological conclusions are highly dependent on the adequacy and reliability of the feature extraction method.

Apart from few special alternate approaches [6,7], all automated methods for feature extraction from isotope-resolved mass spectra compare the observed (experimental) spectral pattern to a set of precalculated theoretical isotope patterns. The calculation of isotope patterns is based on the estimation of average stoichiometries for a particular molecular mass (*averagine*[8] and related methods [9]) or on relative isotope abundance estimation [10] or on protein database-driven mean isotope distribution calculation [11]. The computation of isotope patterns is based on efficient implementations [12-14] of Yergey's original polynomial method [15,16].

Comparison of theoretical and experimental isotope distributions is typically accomplished based on subtractive fitting and peak selection algorithms, attempting to sequentially detect the dominant components in a mixture spectrum. These subset selection methods attempt to determine a small set of basis functions capable of approximating the observed signal well. Facing the infeasibility of an exhaustive search over all possible subsets of explanatory basis functions, they apply greedy search strategies. Here, "greediness" refers to the fact that these approaches consistently overestimate individual feature contributions and are incapable of excluding a basis function once it has been included in the active set. Hence, although providing sparseness, they are not globally optimal. In the context of mixture modeling of mass spectra, these approaches amount to sequential isotope distribution template matching procedures [6,8-11,17-22]. Fitting is carried out via *χ*^2^ distances [8,20], least squares [9-11,17,21-23], weighted least squares [19], or cross-correlation [18,24]. The automatic determination of the charge state associated with an isotope pattern present in an experimental spectrum is based on cross-correlation [19,25] or on dot products in Fourier space [25,26], exploiting the shift theorem of the Fourier transform. There are only few [27] non-greedy feature selection algorithms and mixture model approaches for MS data [28-31]. Among these, *Matching*[28] and Roussis' method [29] rely on manual preselection of contribution candidates. Sparse non-greedy procedures include *pepex*[30] and Du's method [31]. The *pepex* approach is suitable for single charge data and is based on a non-negative sparse regression scheme, with an approximate *L*_0_-norm constraint. Du and Angeletti [31] perform data reduction prior to feature extraction and apply a sparseness-promoting variable selection scheme [32]. With the exception of Du's [31] and Kaur's [19] methods, none of the mentioned mixture model approaches provide support for the detection of a sparse set of *a priori* unknown peptide peaks under an arbitrary set of charge states. Du's method [31] and NITPICK overcome Kaur's greedy iterative weighted least squares fitting approach. In contrast to [31], NITPICK does not rely on a heuristic parameterization and is instead based on statistical model selection, making use of an algorithmically more efficient non-greedy sequential feature selection procedure with a statistically motivated termination criterion. NITPICK was designed to support the calculation of accurate monoisotopic peak lists from raw mass spectra and was specifically tailored to cases where the raw spectra stem from unknown, possibly overlapping experimental isotope patterns of multiple charge states.

The methods section details the mixture modeling approach, fractional averagine for improved stoichiometry estimation and data fitting, and our main contribution, a computationally efficient method for improved non-negative feature selection and the corresponding statistical complexity estimation approach in conjunction with the derivation of a lower bound for early termination. Comparative results on simulated and real-world data sets are given in the results and consequently discussed. Eventually, we conclude and offer perspectives. Derivations of the formulas used in the main article are available in the appendix.

## Methods

**Figure 1:**
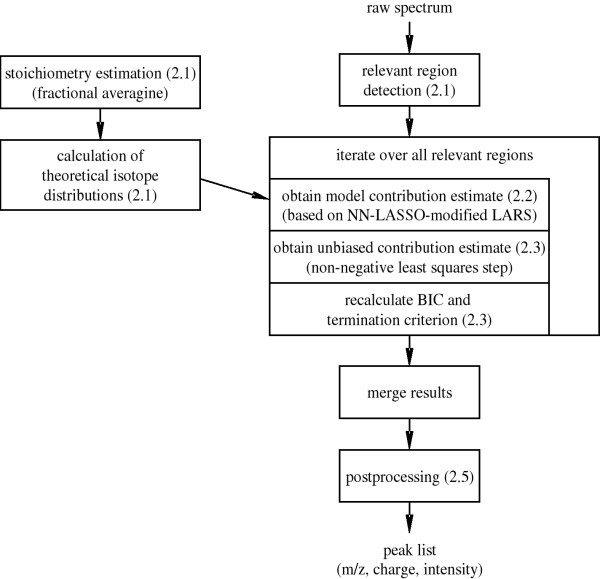
**NITPICK workflow overview.  **Raw spectrum preprocessing, relevant region detection, region-wise peak picking, merging of detected peaks and peak list postprocessing. At the heart of the method lies an iterative feature selection procedure controlled by a statistical termination criterion, as illustrated by the large box in the center. As a tightly interconnected prerequisite to the main workflow, the column on the left depicts the steps required for the calculation of the regression model matrix. Numbers in parentheses give the manuscript sections in which the specific steps are detailed.

The NITPICK algorithm (cf. figure 1) models an observed mixture spectrum as a linear combination of theoretical isotope distribution patterns. Statistically, finding a sensible parameterization of this mixture model amounts to a constrained regression problem in which we seek to minimize the raw signal reconstruction error in a least-squares sense while adhering to a set of additional constraints. Such an approach requires reliable underlying isotope patterns, and we propose an improvement for the well-known averagine model to achieve this goal. We subsequently introduce NITPICK's iterative feature selection procedure, which employs a novel, non-greedy isotope distribution selection method and is based on a statistically motivated termination criterion, attempting to eliminate premature or late iteration termination.

### Mixture model

We assume that observed spectra are available in a discrete (not necessarily equispaced) mass binning scheme defined by a mass vector ***m***= (*m*_1_, *m*_2_, ..., *m*_*N*_)^*T*^ and represent a raw multicomponent mass spectrum by a vector ***s*** of size *N* × 1, where *s*_*i*_ corresponds to the abundance observed in the *i*th mass bin *m*_*i*_. In practical applications, the vector ***s*** may also result from preprocessing steps such as relevant region detection [19] and may thus represent only a part of a complete raw spectrum. The basic assumption behind the mixture model approach is that ***s*** be a linear combination of mass spectrum abundances of *K* pure components *ϕ*_*i*_,

(1) $$s=\sum_{k=1}^{K}c_{i}\phi_{i}=\Phi c.$$

Each of the concentration coefficients *c*_*i*_, *i* = 1, ..., *K* is associated with a column *ϕ*_*i*_ of the *N* × *K* model matrix Φ. We regard these columns as basis functions and their elements *ϕ*_*ji*_ correspond to the mass spectrum abundance expected in the *j*th mass bin *m*_*j*_ of the *i*th pure component *ϕ*_*i*_.

For the estimation of the concentration vector ***c***, the model matrix **Φ** has to be available, and in general this is not the case. One hence resorts to approximating the basis functions by a large set of theoretical isotope distributions (i.e. isotope abundance patterns) densely spread over the prespecified mass/charge binning scheme. Effectively, this recasts the original peak picking task into the framework of a feature (i.e. basis function) selection problem.

#### Model matrix calculation

Given an elemental stoichiometry, the corresponding theoretical isotope distribution is well-defined and can easily be calculated [12-15]. Hence, if a prespecified set of stoichiometries of potential pure components is available, the calculation of the respective set of theoretical isotope distributions (including chemical modifications and multiple charge states) is straightforward. These isotope distributions are subsequently convolved with instrument-specific, possibly mass-dependent peak shape functions, yielding the basis functions *ϕ*_*i*_.

#### Fractional averagine

In many practical applications prior knowledge about potential components is not at hand. Thus, one needs to resort to expected average stoichiometry estimates as a best-effort approximation. In this case, the quality of the feature selection procedure is highly dependent on the quality of the stoichiometry model. We therefore extended the widely used *averagine* approach [8] to amend its discrete and discontinuous nature, gaining models without mass errors and improved true isotope distribution reconstruction properties. *Fractional averagine* provides a real-valued element stoichiometry ***ρ*** = (*ρ_1_,...,ρ*_5_)^*T*^ according to the mapping *f*: ℝ → ℝ^5^ between a mass value and the number of element atoms in an averagine (H_7.75833_C_4.9384_N_1.35777_O_1.4773_S_0.0417_) molecule. The calculation of the theoretical isotope distribution of ***ρ*** is based on the observation that isotope abundances follow a multinomial distribution [33], and that fractional numbers of trials in a multinomial can be modeled as linear interpolation between the probability functions of the multinomials parameterized with the surrounding integers (see appendix A). For computational ease, calculations are carried out in the realm of the corresponding moment generating function (MGF) [34] of the multinomial probability mass function. For the *i*th stoichiometry element, the MGF given *ρ*_*i*_ can be factorized according to

(2) $$\begin{array}{l} M_{x}\left(t_{1},\ldots ,t_{k-1}|\rho_{i}\right)\\ ={\left[p_{1}e^{t_{1}}+\cdots +p_{k-1}e^{t_{k-1}}+p_{k}\right]}^{\left[\rho_{i}\right]+\left(\rho_{i}-\left\lfloor \rho_{i}\right\rfloor \right)}\\ =M_{x^{1}}\left(t_{1},\ldots ,t_{k-1}|\left\lfloor \rho_{i}\right\rfloor \right)M_{x^{2}}\left(t_{1},\ldots ,t_{k-1}|\left(\rho_{i}-\left\lfloor \rho_{i}\right\rfloor \right)\right) \end{array}$$

where *p*_*l*_ is the probability of occurrence of the *l*th isotope $\sum_{l=1}^{k}p_{l}=1$, *x* = (*x*_1_, ..., *x*_*k*_) ^*T*^ denotes the number of times a particular isotope is chosen $\sum_{l=1}^{k}x_{l}=\rho_{i}$ and *t* = (*t*_1_, ..., *t*_*k*_)*^T^* is the corresponding variable of the MGF. By rearrangement of the MGFs of all elements, it is possible to separate integer and real-valued contributions, yielding the common averagine model $\hat{\rho }$ = (⌊*ρ*_1_⌋, ⌊*ρ*_2_⌋, ..., ⌊*ρ*_5_⌋)^*T*^ for the integers and the fractional averagine correction $\tilde{\rho }$ = (*ρ*_1_ - ⌊*ρ*_1_⌋, *ρ*_2_ - ⌊*ρ*_2_⌋, ..., *ρ*_5_ - ⌊*ρ*_5_⌋)^*T*^ for the remaining fractional masses. The theoretical isotope distribution for ${\tilde{\rho }}_{i}$ is given by the linear combination of a peak of intensity one at mass zero and the theoretical isotope distribution of the *i*th averagine element, weighted by 1 - ${\tilde{\rho }}_{i}$ and ${\tilde{\rho }}_{i}$, respectively. Thus, efficient calculation of the theoretical isotope distribution of the stoichiometry $\hat{\rho }$ is carried out based on the Mercury7 algorithm [14], and the theoretical isotope distribution for the fractional stoichiometry ***ρ*** is subsequently obtained with five additional convolution steps.

### Basis function selection

Given the set of basis functions **Φ** = [*ϕ*_1_*ϕ*_2_ ... *ϕ*_*k*_], basis function selection and subsequent determination of the contribution coefficients *c*_*i*_ provides a solution to eq. (1). Thus, as the modeling parameters and, in particular, the monoisotopic masses for all basis function are known, one can determine which isotope distributions are present and in what abundance (assuming ∑_*k*_*ϕ*_*ki*_ = 1).

In practice, basis functions are calculated for each possible monoisotopic mass and each expected charge state, yielding model matrices **Φ** with *KN* (in the case of one basis function per mass/charge bin and charge, we have *K* = *n*_*Z*_*N*, where *n*_*Z*_ corresponds to the number of charge states observable in the experiment; hence, for *n*_*Z*_> 1, there exists an infinite number of solutions for eq. (1)). This is a problem intrinsic to the proposed mixture modeling approach and has been observed previously [23,28,30]. The *least absolute shrinkage and selection operator* (LASSO) [32] enjoys favorable properties of regularization and subset selection. Because the LASSO is capable of shrinking coefficients to exactly zero, it offers a non-greedy way to gain sparse models. The LASSO solution $\hat{c}$ for equation (1) is given by

(3) $$\begin{array}{c} \hat{c}=\arg\underset{c}{\min}\{||s-\Phi c|{|}^{2}\}\\ \begin{array}{cc} \text{s}.\text{t}. & \sum_{i=1}^{K}|c_{i}|\leq t, \end{array} \end{array}$$

where *t* ≥ 0 is a user-defined tuning parameter [31,32]. Mass spectra intensities *s*_*i*_, basis function values *ϕ*_*ji*_, and basis function contributions *c*_*k*_ are strictly non-negative, thus adding a non-negativity constraint to the solution space of $\hat{c}$, yielding

(4) $$\begin{array}{c} \hat{c}=\arg\underset{c}{\min}\{||s-\Phi c|{|}^{2}\}\\ \begin{array}{cc} \text{s}.\text{t}. & \sum_{i=1}^{K}|c_{i}|\leq t,c_{i}\geq 0. \end{array} \end{array}$$

For fixed *t*, this is a quadratic programming problem with linear inequality constraints which can be solved by an active set algorithm, sequentially introducing the inequality constraints and seeking a feasible solution satisfying the Kuhn-Tucker conditions [32,35,36]. Equation (4) corresponds to $\hat{c}(\lambda )=\arg\min_{c}\{||s-\Phi c|{|}^{2}+\lambda \sum_{i=1}^{K}\left|c_{i}\right|\}$ with *c*_*i*_ ≥ 0 where the parameter *t* is related to the Lagrangian multiplier *λ* which determines the number of free parameters *df*(*λ*) in the linear model [32,36-38].

Common procedures for the optimal selection of *λ* or *df*(*λ*) are based on the minimization of the prediction error. This involves estimation of training optimism via *C*_*p*_-statistics, the Akaike Information Criterion (AIC), or the Bayesian Information Criterion (BIC) [37]. Alternatively, direct estimation of prediction error can be carried out via cross-validation or generalized cross-validation (GCV) [37]. All these methods require the LASSO trace $\hat{c}$(*λ*_*l*_), where *λ*_*l*_ ∈ ℒ and $ℒ=\{\lambda_{1},...,\lambda_{\left|ℒ\right|}\}$ defines the set of LASSO regularization parameters for which the prediction error is calculated. In general, the calculation of the LASSO trace is computationally intensive and it is not clear how the elements of ℒ should be selected [36]. *Least angle regression* (LARS) [39] is an algorithmically different approach to variable selection which can be modified such that the LARS algorithm implements the non-negative LASSO from equation (4). The LASSO-modified LARS is a constructive active set procedure which constructs the LASSO regularization path in a stepwise manner. Denote by A(*λ*) the set of indices *i* ∈ {1. ..., *K*} of those *ϕ*_*i*_ which are in the active set for a particular choice of *λ*. Starting from *λ* = ∞ and letting *λ* → 0, the algorithm computes non-negative LASSO solutions for all *λ* for which the active set changes, thus implicitly defining ℒ. The LASSO-modified LARS guarantees A(*λ*_*j*_) ≠ A(*λ*_*j*+1_), but it allows for the deletion of previously selected basis functions, and hence |A(*λ*_*j*_) | need not increase monotonically for increasing *j*. Basis functions can be required to enter the active set in their predefined directions [39] which allows the implementation of a non-negativity constraint. Necessary matrix inversions are constrained to |A(*λ*) | × |A(*λ*)|-sized scatter matrices $\Phi_{A(\lambda )}^{T}\Phi_{A(\lambda )}$ and can be implemented as iterative updates, thus the procedure is computationally efficient.

### Complexity estimation

It is desirable to terminate active set updates as soon as the basis functions in the active set are able to explain the observed data sufficiently well, i.e. until the increase in explanatory power does not justify the increase in model complexity anymore. We now describe a modification to the non-negative LASSO-modified LARS, which enables us to sequentially build a BIC trace along the LASSO regularization path and to identify minima along this trace. Upon termination, the proposed procedure returns the estimate ${\hat{c}}_{A}$ and the set $A=\{i|{\hat{c}}_{A_{i}}>0\}$ of active basis functions.

#### BIC measure

The LARS *C*_*p*_-type risk reestimation formula [39] for optimal selection of *λ* does not hold under the non-negative LASSO modification. Instead, we recalculate a BIC measure

(5) $$\text{BIC}(\lambda )=\frac{1}{\sigma^{2}}\underset{N.\text{MSE}(\lambda )}{\underset{︸}{||s-\Phi_{A(\lambda )}{\hat{c}}_{A(\lambda )}^{q}|{|}^{2}}}+df(\lambda )\log N,$$

in each LARS iteration [40]. For the calculation of the unbiased training error MSE(*λ*) in eq. (5) we require an additional non-negative least squares fit

(6) $$\begin{array}{c} {\hat{c}}_{A(\lambda )}^{q}=\arg\underset{c_{A(\lambda )}}{\min}||s-\Phi_{A(\lambda )}c_{A(\lambda )}|{|}^{2}\\ \begin{array}{cc} \text{s}.\text{ t}. & {\left({\hat{c}}_{A(\lambda )}^{q}\right)}_{i}\geq 0. \end{array} \end{array}$$

The noise variance *σ*^2^ in eq. (5) is estimated as the mean residual sum of squares of a low-bias non-negative least squares estimate [37].

#### Estimation of *df*(*λ*)

The calculation of BIC(*λ*) in eq.(5) requires an estimate for the degrees of freedom *df*(*λ*), which can be obtained via the generalized degrees of freedom (GDF) [38]. The GDF of an NN-LASSO-modified LARS model based on an active set A(*λ*) are given by

(7) $$\text{GDF}(\lambda )=\frac{1}{\sigma^{2}}s^{T}\Phi_{A(\lambda )}{\hat{c}}_{A(\lambda )}^{q}.$$

Because the coefficients ${\left({\hat{c}}_{A(\lambda )}^{q}\right)}_{i}$ > 0 are non-negative, the estimate ${\hat{c}}_{A(\lambda )}^{q}$ solves

(8) $${\hat{c}}_{A(\lambda )}^{q}=\arg\underset{{\hat{c}}_{A(\lambda )}^{q}}{\min}\left\{||s-\Phi c_{A(\lambda )}^{q}|{|}^{2}+\lambda \sum_{i=1}^{K}{\left(c_{A(\lambda )}^{q}\right)}_{i}\right\}$$

which is differentiable with respect to ${\left({\hat{c}}_{A(\lambda )}^{q}\right)}_{i}$. Setting the derivative to zero, we obtain

(9) $${\hat{c}}_{A(\lambda )}^{q}={\left(\Phi_{A(\lambda )}^{T}\Phi_{A(\lambda )}\right)}^{-1}(\Phi_{A(\lambda )}^{T}s-\frac{1}{2}\lambda 1_{A(\lambda )}).$$

Hence, given an active set A(*λ*), the generalized degrees of freedom from eq. (7) can be written as

(10) $$\begin{array}{l} \text{GDF}(\lambda )\\ =s^{T}\frac{1}{\sigma^{2}}(\Phi_{A(\lambda )}{\hat{c}}_{A(\lambda )}^{q})\\ =s^{T}\frac{1}{\sigma^{2}}\Phi_{A(\lambda )}{\left(\Phi_{A(\lambda )}^{T}\Phi_{A(\lambda )}\right)}^{-1}(\Phi_{A(\lambda )}^{T}s-\frac{1}{2}\lambda 1_{A(\lambda )}) \end{array}$$

which is monotonously increasing for decreasing *λ* (see appendix B for a proof).

#### Optimal termination

**Figure 2:**
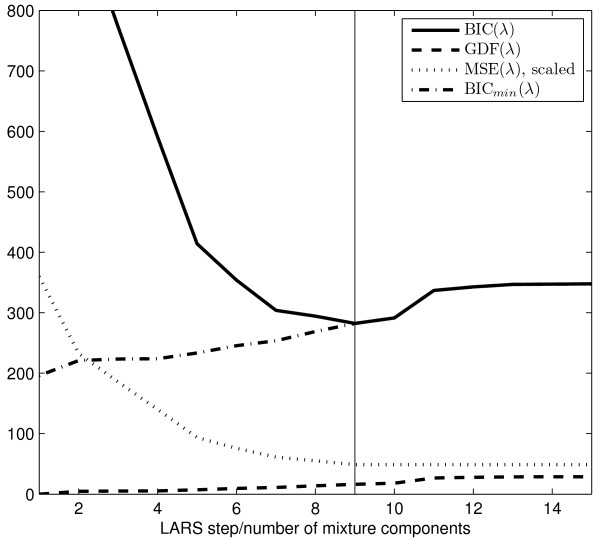
**Efficient automated determination of the number of components in an area with overlapping peaks using the BIC*_min_(λ)* termination criterion. ** The mean squared error MSE (scaled, dotted) decreases monotonically over the LARS steps and the generalized degrees of freedom GDF*(λ)* (dashed) increase monotonically.  The resulting BIC*(λ)* measure (solid) exhibits a minimum BIC(*λ_9_*) in the 9th LARS step and *λ_9_* is accepted as a minimizer because the lower bound BIC*_min_(λ_10_)* exceeds BIC(*λ_9_*) in the 10th LARS step.

The minimal possible training error of the model is attained when all variables are in the active set, in which case the respective coefficients ${\hat{c}}^{q}$ are given by ${\hat{c}}^{q}$ = arg min_*c*_ ||**s** - **Φ*c***||^2^ subject to *c*_*i*_ ≥ 0, and the corresponding error is $\text{MSE}=\frac{1}{N}||s-\Phi {\hat{c}}^{q}|{|}^{2}$. Thus, a lower bound for BIC(*λ*) is given by

(11) $${\text{BIC}}_{min}(\lambda )=\frac{N}{\sigma^{2}}\text{MSE}+\text{GDF}(\lambda )\log N$$

(see appendix C for a proof). In general, BIC(*λ*) will have several minima for increasing values of GDF(*λ*), hence we track the minimum BIC(*λ*_*min*_) through the NN-LASSO-modified LARS cycles and accept *λ*_*min*_ as a minimizer as soon as the lower bound BIC_*min*_(*λ*) of a subsequent LARS step exceeds the current best estimate BIC(*λ*_*min*_), i.e. BIC(*λ*_*min*_) < BIC_*min*_(*λ*) (see figure 2).

### Regression on selected models

The sum constraint in equation (4) is ultimately responsible for the sparseness property of the LASSO. Its regularizing effect is similar to the one of the regularization term found in ridge regression, especially with respect to the fact that all LASSO estimates ${\hat{c}}_{i}$, *i* = 1, ..., *K* are subject to shrinkage [32,37] and represent biased versions of the least squares estimates. Given an active set A, the shrinkage bias on the ${\hat{c}}_{i}$ can effectively be removed by introducing a subsequent non-negative least squares regression step after the basis functions have been selected by the LASSO procedure [32]. This also holds true for the NN-LASSO-modified LARS procedure, and the corresponding unbiased quantification estimate ${\hat{c}}_{A}^{q}$ is given by equation (6) with A(*λ*) = A.

## Postprocessing

The estimate $\hat{c}$ is subject to modeling errors and these shortcomings lead to suboptimal NN-LASSO-modified LARS estimates and active sets. In particular, the estimation depends on the match between the observed and theoretical peak shape function. Especially in high mass resolution experiments, one can frequently observe spurious peak detections in bins directly adjacent to monoisotopic mass bins of true peaks [30]. A possible remedy is a local maximum detection implemented as a postprocessing filter *φ*(·) applied to the active basis function index set A:

(12) $$\begin{array}{c} A^{\prime }=\varphi (A|G)\\ =\{j\in A|{\left({\hat{c}}_{A}^{q}\right)}_{j}=\max\{{\left({\hat{c}}_{A}^{q}\right)}_{l}|l\in \nu_{G}(j)\}\} \end{array}$$

where $\nu_{G}(j)=\{k\in A||b_{k}-b_{j}|\leq \frac{G-1}{2}\}$ defines an *m/z*-neighborhood of size *G* around each peak and *b*_*j*_ is the mass/charge bin index of the monoisotopic mass *m*_0_ of the *j*th theoretical isotope distribution *ϕ*_*j*_. If $A\neq A^{\prime }$, ${\hat{c}}_{A(\lambda )}^{q}$ is reestimated using eq. (6) with $A(\lambda )=A^{\prime }$

## Results

### Stoichiometry models

The fractional averagine stoichiometry model was compared against the classical averagine model based on the analysis of their respective approximation errors using simulated theoretical peptide isotope distributions.

#### Data Set

All UniProt (version 51.4.) [41] human proteins were subjected to *in silico* tryptic digestion. For each of the *R* digestion product peptides $P_{r},r\in \{1,...,R\text{\}}$, exact element stoichiometries $\rho_{r}^{x}$ and exact theoretical isotope distributions $d_{r}^{x}$ were calculated. Peptides with monoisotopic masses above *m/z* 5000 were discarded.

## Comparison of deviations

**Figure 3:**
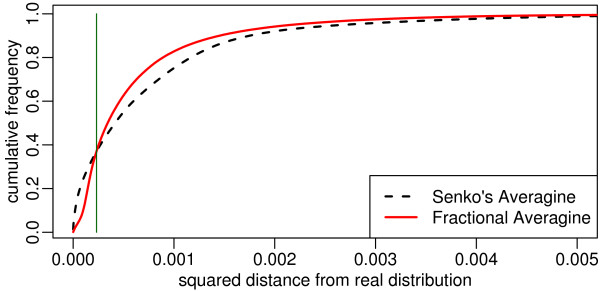
**Comparison of the impact of averagine and fractional averagine stoichiometry estimation errors on the estimation of theoretical isotope distributions.  ** The cumulative histograms of least squares deviations from the true theoretical isotope distribution illustrate the superior overall performance of fractional averagine (solid line) compared to Senko's classical averagine (dashed line):  fractional averagine causes a 17% decrease in mean squared error magnitude.

Classical and fractional averagine were used to estimate approximate element stoichiometries ${\hat{\rho }}_{r}$ and $\rho_{r}$, respectively, for all peptides $P_{r}$ in the data set. Based on ${\hat{\rho }}_{r}$ and $\rho_{r}$, the corresponding theoretical isotope distribution intensity vectors ${\hat{d}}_{r}$ and $d_{r}$ were calculated. Figure 3 shows the cumulative distribution of the squared differences between the classical averagine and the true theoretical isotope distribution intensity vectors ($||{\hat{d}}_{r}-d_{r}^{x}|{|}_{2}^{2}$, dashed black), and fractional averagine and the true theoretical isotope distribution intensity vectors ($||d_{r}-d_{r}^{x}|{|}_{2}^{2}$, solid red).

## Peak picking

For peak picking/feature extraction performance evaluation, we determine representative peak picking statistics: we calculate accuracy, sensitivity, specificity, and positive and negative predictive values on simulation data. Further, and in contrast to previous contributions, we explicitly perform manual *validation* on a real-world data set.

### Data sets

#### Simulation data sets

For the simulation, all UniProt (version 51.4.) [41] human protein sequences were subjected to *in silico* tryptic digestion. Simulation sets were generated by random drawing of digestion product peptides and intensities. To ensure a fair comparison with the pepex procedure (which was selected for benchmarking as the only publicly available procedure implementing non-greedy feature extraction) which is limited to singly charged data sets, all simulated peptide were endowed with a single charge. *Mercury7 *[14] was used for the calculation of the respective theoretical isotopic distributions. After convolution with an *m/z*-dependent Gaussian aperture function [42], intensity-weighted linear combinations of peptide spectra were calculated and a Poisson noise model (see appendix D) was applied to obtain spectra of different signal to noise (SNR) ratios. Simulations were performed in the densely populated *m/z* 500–700 range (see Additional file 1 for the data sets).

#### Real-world data set

Experiments on real-world data were performed using Bovine Serum Albumin (BSA) LC/(ESI-)MS calibration data. The data set was acquired on a QSTAR XL mass spectrometer (Applied Biosystems/MDS Sciex) equipped with microsale capillary HPLC system (Famos Autosampler, LC packings, Agilent 1100 HPLC pump). A mixture spectrum with many overlapping peaks was obtained by integration of the LC/MS data set over the retention time domain (see Additional files 2 and 3). Peak identification was carried out in the *m/z* 500–700 range and peak shape functions were modeled according to mass-dependent Gaussian distributions with standard deviations *σ*(*m/z*) = 0.005 *m/z *[42].

## Performance estimation

We characterize peak picking performance based on a set of measures from statistical test theory, all of which depend on the availability of the numbers of true positives (TP), true negatives (TN), false positives (FP) and false negatives (FN).

Ground truth is based on knowledge of the complete set of peptide signals present in a mass spectrum. For simulated data sets, this information is available. In real-world experiments, the definition of ground truth is complicated by sample complexity, stochastic sample modification, non-peptidic components and limited dynamic range. As a consequence, TNs, FNs and the overall number of true peaks are not available for real-world data, limiting the available statistical measures to positive predictive values and the ratio of true positives (sensitivity ratios).

Nevertheless, we can determine the number of TPs and FPs in both cases: we check whether a detected peak really exists and if it has been assigned its correct monoisotopic mass *m*_0 _and charge *z*. If so, it is counted as true positive (TP) or, otherwise, as false positive (FP).

### Simulation data

As the complete set of simulated peaks is known, the remaining set of undetected peaks can be determined and its members are counted as false negatives (FN). With the true number of positives and negatives available the calculation of the number of true negatives (TN) is straightforward, thus enabling the use of related statistical test error measures for performance characterization:

• accuracy (ACC) measures the rate of correct peak vs. no peak decisions, i.e. $\text{ACC}=\frac{\text{TP}+\text{TN}}{\text{TN}+\text{FP}+\text{TP}+\text{FN}}$

• the negative predictive value (NPV) gives the rate at which there is no peak at positions where the procedure was unable to find a peak, $\text{NPV}=\frac{\text{TN}}{\text{TN}+\text{FN}}$

• the positive predictive value (PPV) measures the rate of correct peak detections among all peaks detected by the procedure, $\text{PPV}=\frac{\text{TP}}{\text{TP}+\text{FP}}$

• sensitivity (SE) measures the method's ability to detect a peak if it exists, $\text{SE}=\frac{\text{TP}}{\text{TP}+\text{FN}}$

• specificity (SP) measures the method's ability to correctly identify the absence of peaks in the spectrum, $\text{SP}=\frac{\text{TN}}{\text{TN}+\text{FP}}$

All measures have been computed with and without the application of postprocessing.

### Real-world data

Resorting to LC/MS data and creating a semi-artificial data set by integration over the retention time domain was motivated by the fact that this approach yields a data set accessible to human manual validation. With LC resolution power available to the human expert (and resorting to comparatively simple mixtures), all peaks detected in the integrated mixture can still be manually verified. Exemplary peak picking results are illustrated below.

## Comparative results

### Pepex

We chose to compare NITPICK to a conceptually similar, model-based approach called pepex [30]. In contrast to model-free approaches and in accordance with NITPICK, pepex models observed spectra based on a linear mixture model, which is augmented by a complexity constraint. It uses the averagine model to describe unknown features and is capable of terminating its feature selection routine after a sufficient number of basis functions has been selected. However, as the publicly available implementation of the pepex approach is limited to charge state *z *= 1 data sets, NITPICK comparison against pepex was limited to the simulated data set.

For the analysis, the pepex algorithm was tailored to the problem at hand: its parameters were heavily optimized to maximize peak picking performance on the simulation data set. As a consequence, the reported results underestimate the pepex generalization error and overestimate its performance (see Additional file 4). For NITPICK, no specific parameter optimization was carried out, postprocessing was kept to a minimum (*G *= 3), and the reported results are representative (see Additional file 5).

## MarkerView

We also compared NITPICK's ability to extract peak information from a retention time integrated mixture spectrum against the proprietary MarkerView application (Applied Biosystems/MDS Sciex, Concord, Canada) version 1.2, which includes an LC/MS peak picking algorithm. In contrast to NITPICK, MarkerView was provided with the original LC/MS data set and thus had retention time information available. Peak picking was carried out in the *m/z *400–1400 range and detected peaks were manually validated (see Additional files 6 and 7).

## Discussion

### Stoichiometry models

In comparison (see figure 3, classical averagine in dashed black, fractional averagine in solid red), Senko's classical averagine [25] features a larger number of very small deviances from the truth than fractional averagine. This is caused by the rounding to integers property of the classical approach, yielding exact models more often. At the same time, the deviance distribution of the fractional averagine model has a significantly lighter tail, i.e. the model generates significantly less stoichiometries whose theoretical isotope distributions have large deviations. The cumulative distribution based on the fractional averagine model approaches 1 more quickly, and its use yields an overall decrease in theoretical isotope distribution deviations. This finding is supported by the corresponding one-sided non-parametric Mann-Whitney test (*p *< 2.6 × 10^-11^). Because the overall impact on the peak picking performance depends on the squared mean error magnitude (7.6 × 10^-4 ^for classical averagine, 6.3 × 10^-4 ^for fractional averagine, corresponding to a 17% decrease for fractional averagine), fractional averagine clearly is the preferable model.

### Peak picking

#### Simulation data set

**Figure 4:**
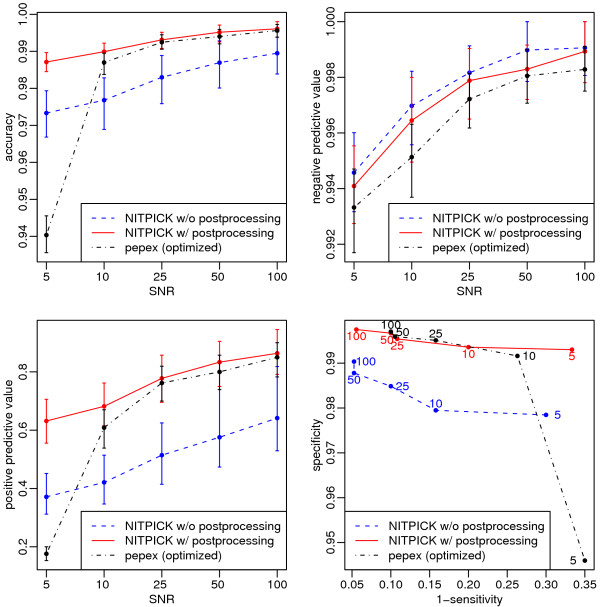
**Evaluation and comparison with the pepex algorithm on simulated data.  ** Accuracy (top left), negative predictive values (top right), positive predictive values (bottom left) and sensitivity-specificity traces (bottom right).  Plots show NITPICK results in solid red, NITPICK results without postprocessing in dashed blue and pepex results (optimized, see text) in dashed-dotted black. NITPICK is clearly superior in terms of accuracy, specificity and sensitivity.

Figure 4 shows the results for the peak detection performance analysis. As expected, ACC, NPV and PPV improve with increasing SNR. Postprocessing causes a decrease in NPV and an increase in PPV for all SNR levels as the removal of spurious peaks decreases FP but also, erroneously, increases FN. The ACC plot (top left) illustrates the fact that NITPICK is successful at simultaneously maximizing PPV and NPV. Postprocessing can then be used to trade specificity for sensitivity as supported by the sensitivity-specificity trace in figure 4 (bottom right). Here, each dot marks sensitivity and specificity of a given NITPICK postprocessing parameterization. Lines connect points of different SNRs. As expected, the introduction of a postprocessing step increases specificity and decreases sensitivity. Further analysis of FNs in the simulated data reveals that false negatives are predominantly due to low-intensity components in complex mixtures (data not shown).

#### Comparative results

In comparison with pepex, NITPICK exhibits better results with respect to all statistical measures in figure 4. It is especially obvious that pepex suffers from a severe increase in false positives (FPs) for very low SNR situations, yielding significant decreases in accuracy (ACC) and specificity (SP). For PPV, although the pepex approach outperforms NITPICK when no postprocessing is applied, it is inferior to the full NITPICK algorithm with simple spurious peak removal corresponding to eq. (12). With respect to sensitivity (SE) and specificity (SP), figure 4 reveals constant high (above 0.99) and superior specificity values for NITPICK at greatly increased sensitivity. Thus one can conclude that the NITPICK algorithm is more sensitive than pepex and, at the same time, provides picked peaks with higher confidence.

#### Real-world data set

**Figure 5:**
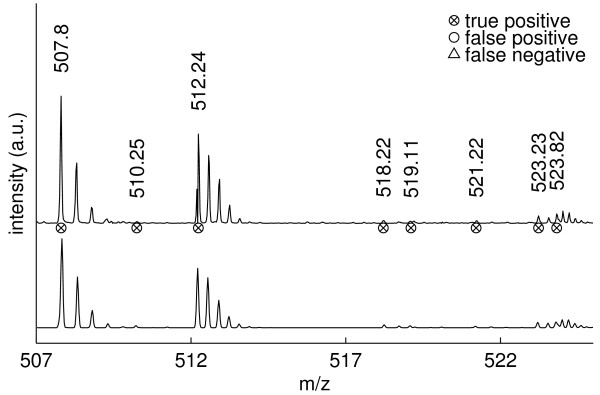
**Peak picking in the *m/z* 507-525 mass range.  ** Illustration of observed (top) and reconstructed (bottom) spectra.  All detected peaks could be confirmed, including the monoisotopic masses of the mixture distribution with components located at *m/z* 523.23 (*z*=3) and *m/z* 523.82 (*z*=5).

We give peak picking illustrations for the mass ranges *m/z *507–525 (with a zoom on *m/z *518–525), *m/z *636–646, *m/z *695–725 and *m/z *775–782, detailing positive and negative peak picking performance aspects. In the *m/z *507–525 mass range (figure 5), all picked peaks could be verified, including the monoisotopic masses of the mixture distribution with components located at *m/z *523.23 (*z*=3) and *m/z *523.82 (*z*=5). Upon re-examination of the raw data, we detected a missed low-intensity peak at *m/z *515.76.

**Figure 6:**
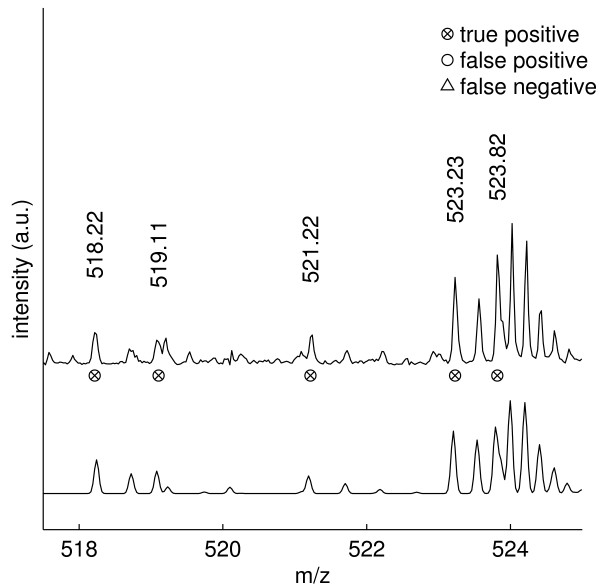
**Zoom on the *m/z* 518–525 mass range.  ** NITPICK proves capable of resolving overlapping isotope distributions and assigning correct monoisotopic masses for the distributions located at *m/z* 518.22 and 519.11 and at *m/z* 523.23 and 523.82.  See text for details.

Figure 6 zooms onto two cases of overlapping isotope distributions in the *m/z *518–525 mass range. At *m/z *518.22 and *m/z *519.11 NITPICK resolves two distinct monoisotopic masses, in spite of their unfavorable mass distance. Although the second isotope peak of the doubly charged ion with monoisotopic mass *m/z *518.22 exhibits a heavy overlap with the monoisotopic peak of the ion at *m/z *519.11, NITPICK is still able to correctly detect the monoisotopic peaks of the two isotope distributions. NITPICK also separates two isotope distributions located at *m/z *523.23 (*z*=3) and *m/z *523.82 (*z*=4). The detection of the monoisotopic mass at *m/z *523.82 is particularly non-trivial because of its heavy overlap with an isotope peak of the isotope distribution located at *m/z *523.23 and also because the detected monoisotopic mass peak at *m/z *523.82 is not the most abundant peak within its isotope distribution.

**Figure 7:**
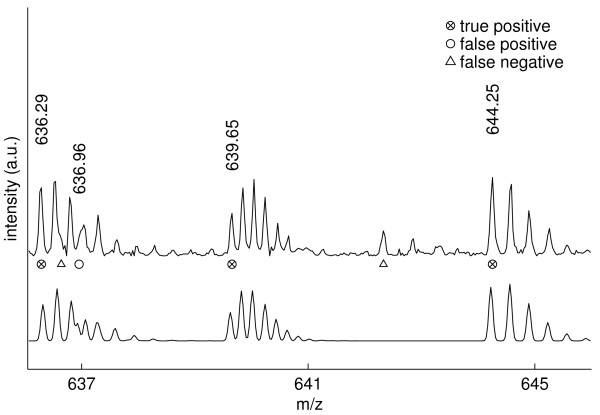
**Peak picking results in the *m/z * 636–646 mass range.  **Illustration of observed (top) and reconstructed (bottom) spectra. At *m/z* 636.64 and *m/z* 636.96 we observe incomplete unmixing: The isotope distribution *(z=3)* with monoisotopic mass m0 located at *m/z* 636.29 heavily overlaps the distribution *(z=3)* with m0 = 636.64 *m/z* (left triangle marker). The overlap proves inseparable and the monoisotopic mass of the second distribution is wrongly detected at *m/z* 636.96. Further, due to conservative noise level/complexity estimation, the isotope distribution located at *m/z* 642.33 (right triangle marker) is not detected. Note that in both of the distributions located at *m/z* 636.29 and *m/z* 639.65, the monoisotopic mass peak does not correspond to the most intensive peak.

In the *m/z *636–646 mass range (figure 7) we observe an example of incomplete unmixing: the isotope distribution (*z*=3) with monoisotopic mass located at *m/z *636.29 heavily overlaps the distribution (*z*=3) located at *m/z *636.64 (left triangle marker). The overlap proves inseparable and the monoisotopic mass of the second distribution is wrongly detected at *m/z *636.96. Further, due to conservative noise level/complexity estimation, the isotope distribution located at *m/z *642.33 (right triangle marker) is not detected. Note that in both of the correctly detected distributions located at *m/z *636.29 and *m/z *639.65, the monoisotopic mass peak does not correspond to the most prominent peak.

**Figure 8:**
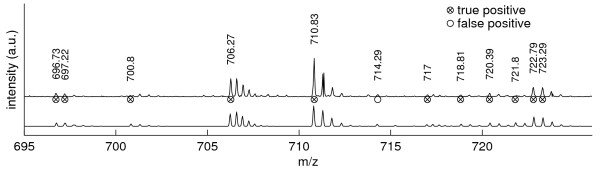
**Peak picking in the *m/z* 695–725 mass range.  ** Illustration of observed (top) and reconstructed (bottom) spectra.  With a single exception, all detected peaks could be manually confirmed. The peak detected at *m/z* 714.29 corresponds to the first isotope peak of the isotope distribution located at *m/z* 713.78 (*z*=2). In the *m/z* 718–724 region the algorithm proves capable of resolving nontrivial low-intensity mixtures.

In the *m/z *695–725 mass range (figure 8), with one exception, all detected peaks could be verified. The wrongly detected peak at *m/z *714.29 corresponds to the first isotope peak of the isotope distribution located at *m/z *713.78 (*z *= 2). Especially in the *m/z* 718 to *m/z *724 region the algorithm proves capable of resolving nontrivial low-intensity mixtures.

**Figure 9:**
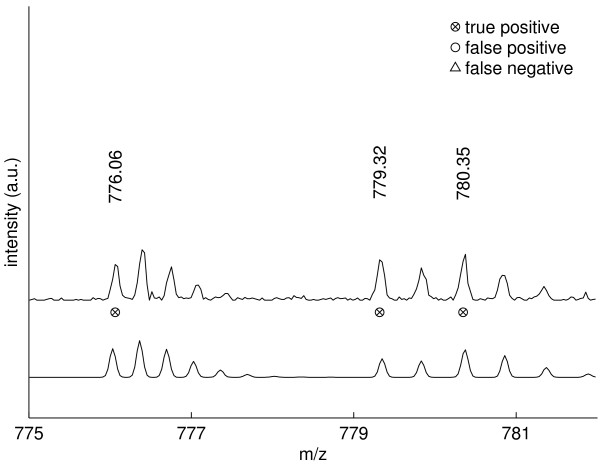
**Observed (top) and reconstructed (bottom) mass spectrum in the *m/z* 775–782 range.  ** The separation of two heavily overlapping isotope distribution clearly illustrates the benefits of NITPICK's intensity model-based approach to the peak picking/feature extraction problem:  the second isotope peak of the isotope distribution located at *m/z * 779.32 (charge 2) and the monoisotopic peak of the distribution located at *m/z * 780.35 (charge 2) are exactly superimposed and can only be distinguished by taking intensity information into account.

In the *m/z *775–782 range (figure 9), the separation of two heavily overlapping isotope distribution clearly illustrates the benefits of NITPICK's intensity model-based approach to the peak picking/feature extraction problem: the second isotope peak of the isotope distribution located at *m/z *779.32 (*z*=2) and the monoisotopic peak of the distribution located at *m/z *780.35 (*z*=2) overlap completely and can only be distinguished by taking intensity information into account.

Overall, the results obtained on real-world data are in agreement with simulation results: after manual validation of 192 peaks detected in the real-world dataset, we observe 127 true positives, yielding a positive predictive value of PPV = 66.15%.

#### Comparison with MarkerView

On the BSA data set, MarkerView detected 388 peaks, for 96 (24.7%) of which charge state information was available. Peaks without charge state assignment were counted as true peaks if their detected mass/charge ratio was correct. This resulted in 205 true positives for 82 (40.0%) of which charge state information was available. In comparison to NITPICK, this yields a sensitivity ratio of $\text{SER}=\frac{{\text{SE}}_{Mar\ker View}}{{\text{SE}}_{NITPICK}}=\frac{205}{127}=1.61$ and a positive predictive value of PPV = 0.53.

As expected, with retention time information available, MarkerView manages to detect a significantly larger number of peaks. Surprisingly, though, retention time information did not contribute to an increased PPV. The partial lack of charge state information also caused the performance interpretation to favor MarkerView: for peaks with correct mass/charge ratio, we assumed completely error-free charge state assignments, which is unlikely to hold true in reality. In contrast, in absence of retention time information, NITPICK delivered charge state information for each and every peak and peaks were counted as true positives if and only if their assigned charge state was correct. MarkerView's PPV and SER are subject to overestimation, whereas NITPICK's PPV is not. Even under this pro-MarkerView bias, if joint maximization of PPV and sensitivity is desired, NITPICK arguably proved competitive with MarkerView: despite the 1.6-fold increase in sensitivity, only slightly more than half of the peaks reported by MarkerView are true positives.

Analysis CPU time on the real-world spectrum was 114s on a 2 GHz AMD Opteron machine. Measurements are based on native, interpreted R code. Preliminary tests with an in-house C++ implementation (to be published elsewhere) yielded a speed increase by a factor of ≈ 20.

## Conclusion and perspectives

### Conclusions

We present NITPICK, an iterative, non-greedy, globally optimal mixture modeling approach for feature extraction from multicomponent mass spectra. The calculation of the set of explanatory theoretical isotope distributions is based on *fractional averagine*, a mass error-free extension to the well-known *averagine *[8] model. Subsequent feature selection is driven by a modified *least angle regression *[8] algorithm for which we derived a suitable, statistically motivated early stopping criterion. Experiments show that NITPICK is able to unmix and deconvolve complex mixture mass spectra. The algorithm was thoroughly evaluated on simulated and real-world data sets and was found to perform better than a conceptually similar algorithm. NITPICK was even found to deliver competitive results when compared against a vendor-supplied algorithm which, in contrast to NITPICK, had retention time resolution available.

We would like to note that although the analysis at hand was confined to a proteomics data set, the application of the proposed methodology is in no way limited to this type of data and can easily be adapted to similar problems outside the field of proteomics.

NITPICK is available as software package for the R programming language and can be downloaded from .

### Perspectives

The constrained least squares regression model in equation (3) implicitly assumes Gaussian noise on the observed spectra. Especially with low-intensity time-of-flight spectra the Gaussian approximation is crude, yielding suboptimal estimates. The incorporation of a data type- and intensity-dependent procedure pursuing a suitable Poisson regression approach [36] in appropriate cases could improve on this shortcoming.

The non-negative least squares step in equation (6) assumes error-free basis functions *ϕ*_*i*_. Although fractional averagine improves over the classical averagine model, this assumption is still violated. Possible remedies include direct intensity estimation techniques [43,44] and enhanced sparse feature selection methodology which allows for errors in explanatory variables. Alternatively, extended stoichiometry models could provide problem-tailored basis functions if model bias is not an issue.

For charge states *z *< 3 and mass ranges *m/z *≲ 1400, there exist so-called *forbidden regions *[45] within the mass spectrum, i.e. mass ranges which are inaccessible to peptides (including modifications). Such information has been reported to be suitable as a preprocessing filter [31].

Further computational efficiency could be achieved by a complexity-driven hierarchical estimation approach, resorting to subtractive feature extraction for simple signals and to the full mixture modeling for complex samples only.

## Appendix

### A Computation of fractional averagine

For the computation of the isotopic distribution of fractional averagine, we build on the fact that the distribution of the isotopes of an element follows a multinomial [33]. The multinomial is discrete, hence for fractional counts of events we can interpolate between the two adjacent integer multinomials for each element such that

(13) $$\begin{array}{l} P_{n=c}(X_{1}=x_{1},...,X_{k-1}=x_{k-1})\\ =\left(\left\lceil c\right\rceil -c\right)P_{n=\left\lfloor c\right\rfloor }\left(X_{1}=x_{1},...,X_{k-1}=x_{k-1}\right)\\ +\left(c-\left\lfloor c\right\rfloor \right)P_{n=\left\lceil c\right\rceil }\left(X_{1}=x_{1},...,X_{k-1}=x_{k-1}\right) \end{array}$$

with ⌈*c*⌉ = min_*j*∈ℕ_(*j *≥ *c*), ⌊*c*⌋ = max_*j*∈ℕ_(*j *≤ *c*) and *X*_*i *_representing the number of times the *i*th isotope of an element occurs. Under the (reasonable) assumption of independence of the atomic distributions of the elements, the resulting joint distribution for a molecule follows from the multiplication of the distributions of its elements.

By changing the order of multiplication and separating the highest possible integer number from the remaining fractional numbers, the calculation of fractional averagine can be related to the Mercury7 algorithm [14], yielding a highly efficient calculation scheme (see eq. (2)). For the convolution of the Mercury integer results and the fractionals we follow [46]: Let *g*_*p*_(*i*) represent the *i*th element of the probability vector of the first and *f*_*p*_(*j*) the *j*th element of the second distribution, then

(14) $$h_{p}(k)=\sum_{i}g_{p}(i)f_{p}(k-i)$$

can be used to compute *h*_*p*_(*k*), the *k*th element of the new vector of probabilities for the joint distribution. Similarly, the corresponding mass vector *h*_*m *_can be computed using the probability vectors *g*_*p *_and *f*_*p *_and the corresponding mass vectors *g*_*m *_and *f*_*m *_using

(15) $$\begin{array}{c} h_{m}(k)={\left(\sum_{i}g_{p}(i)f_{p}(k-i)\right)}^{-1}\\ \sum_{i}g_{p}(i)f_{p}(k-i)(g_{m}(i)+f_{m}(k-i)). \end{array}$$

### B Proof of the monotony of the GDF for the non-negative lasso

As long as a given set $\Phi_{A(\lambda )}$ is valid, it can be easily shown that the GDF are monotonous in *λ*. Starting with the *GDF*(*λ*) of equation (10),

(16) $$\begin{array}{l} \text{GDF}(\lambda )\\ =s^{T}\frac{1}{\sigma^{2}}\Phi_{A(\lambda )}{\left(\Phi_{A(\lambda )}^{T}\Phi_{A(\lambda )}\right)}^{-1}\left(\Phi_{A(\lambda )}^{T}-\frac{1}{2}\lambda 1_{A(\lambda )}\right)\\ =\frac{1}{\sigma^{2}}\sum_{i=1}^{N}s_{i}\\ {\left(\Phi_{A(\lambda )}{\left(\Phi_{A(\lambda )}^{T}\Phi_{A(\lambda )}\right)}^{-1}\left(\Phi_{A(\lambda )}^{T}s-\frac{1}{2}\lambda 1_{A(\lambda )}\right)\right)}_{i}\\ =\frac{1}{\sigma^{2}}\sum_{i=1}^{N}s_{i}{\left(\Phi_{A(\lambda )}{\left(\Phi_{A(\lambda )}^{T}\Phi_{A(\lambda )}\right)}^{-1}\Phi_{A(\lambda )}^{T}s\right)}_{i}\\ -\lambda \frac{1}{2\sigma^{2}}\sum_{i=1}^{N}s_{i}{\left(\Phi_{A(\lambda )}{\left(\Phi_{A(\lambda )}^{T}\Phi_{A(\lambda )}\right)}^{-1}1_{A(\lambda )}\right)}_{i} \end{array}$$

To show that the GDF are monotonously increasing for decreasing values of *λ*, it suffices to analyze the following part of the formula,

(17) $$\begin{array}{l} \sum_{i=1}^{N}\left(s_{i}{\left(\Phi_{A(\lambda )}{\left(\Phi_{A(\lambda )}^{T}\Phi_{A(\lambda )}\right)}^{-1}1_{A(\lambda )}\right)}_{i}\right)\\ =\sum_{i=1}^{N}{\left(e_{i}^{T}\left(\Phi_{A(\lambda )}{\left(\Phi_{A(\lambda )}^{T}\Phi_{A(\lambda )}\right)}^{-1}1_{A(\lambda )}\right)\right)}^{T}(e_{i}^{T}s)\\ =\sum_{i=1}^{N}\left(1_{A(\lambda )}^{T}{\left(\Phi_{A(\lambda )}^{T}\Phi_{A(\lambda )}\right)}^{-1}\Phi_{A(\lambda )}^{T}\right)e_{i}e_{i}^{T}s\\ =\left(1_{A(\lambda )}^{T}{\left(\Phi_{A(\lambda )}^{T}\Phi_{A(\lambda )}\right)}^{-1}\Phi_{A(\lambda )}^{T}\right)I_{N}s\\ =\left(1_{A(\lambda )}^{T}{\left(\Phi_{A(\lambda )}^{T}\Phi_{A(\lambda )}\right)}^{-1}\Phi_{A(\lambda )}^{T}\right)s\\ =\sum_{j=1}^{K}{\hat{c}}_{j}^{LS_{A(\lambda )}} \end{array}$$

where *e*_*i *_denotes the *i*th canonical unit vector of length *N *and $I_{N}=\sum_{i=1}^{N}e_{i}e_{i}^{T}$ is the identity matrix of size *N*.

${\hat{c}}_{j}^{LS_{A(\lambda )}}$ is the least squares regression coefficient for the corresponding least squares problem of the active set. It is known that all non-negative lasso coefficients ${\hat{c}}_{A{\left(\lambda \right)}_{j}}^{q}$ are greater or equal zero, so

(18) $$\begin{array}{l} \sum_{j=1}^{K}{\hat{c}}_{A{\left(\lambda \right)}_{j}}^{q}\geq 0\\ \overset{\left(eq.\;9\right)}{\underset{}{\Leftrightarrow }}\left(1_{A(\lambda )}^{T}{\left(\Phi_{A(\lambda )}^{T}\Phi_{A(\lambda )}\right)}^{-1}\Phi_{A(\lambda )}^{T}\right)s\\ -1_{A(\lambda )}^{T}{\left(\Phi_{A(\lambda )}^{T}\Phi_{A(\lambda )}\right)}^{-1}\frac{1}{2}\lambda 1_{A(\lambda )}\geq 0\\ \Leftrightarrow \left(1_{A(\lambda )}^{T}{\left(\Phi_{A(\lambda )}^{T}\Phi_{A(\lambda )}\right)}^{-1}\Phi_{A(\lambda )}^{T}\right)s\\ \geq \frac{1}{2}\lambda 1_{A(\lambda )}^{T}{\left(\Phi_{A(\lambda )}^{T}\Phi_{A(\lambda )}\right)}^{-1}1_{A(\lambda )}\geq 0\\ \Leftrightarrow \sum_{j=1}^{K}{\hat{c}}_{j}^{LS_{A(\lambda )}}\\ \geq \frac{1}{2}\lambda 1_{A(\lambda )}^{T}{\left(\Phi_{A(\lambda )}^{T}\Phi_{A(\lambda )}\right)}^{-1}1_{A(\lambda )}\geq 0 \end{array}$$

as $\Phi_{A(\lambda )}^{T}\Phi_{A(\lambda )}$ is the inverse of a covariance matrix and, thus, positive-semidefinite, and *λ *is by definition always greater or equal 0. Thus, the second part of equation (16) is monotone with regard to *λ *and therefore the GDFs are monotone as long as a given active set is valid.

It remains to be shown that changes of $\Phi_{A(\lambda )}$ do not influence the monotony, so it needs to be shown that neither the addition of *ϕ*_*j *_to the set $\Phi_{A(\lambda )}$ nor the removal of *ϕ*_*k *_from $\Phi_{A(\lambda )}$ lead to a decrease of cov(***s***, $\Phi_{A(\lambda )}{\hat{c}}_{A(\lambda )}^{q}$) as given in (10). A formal proof is given further below, nevertheless, this can also be argued intuitively.

In the non-negative LARS implementation as described above and in [39], a variable *ϕ*_*j *_will be added to the active set $\phi_{A(\lambda )}$ only if it is positively correlated with the remaining residuals, i. e. if

(19) $$\mathrm{cov}\left(\phi_{j},s-\Phi_{A(\lambda )}{\hat{c}}_{A(\lambda )}^{q}\right)>0$$

This obviously leads to an increase of cov (***s***, $\Phi_{A(\lambda )}{\hat{c}}_{A(\lambda )}^{q}$) as less unexplained variation remains. A variable *ϕ*_*k *_is removed from the active set $\Phi_{A(\lambda )}$ only if cov (*ϕ*_*k*_, ***s ***- $\Phi_{A(\lambda )}{\hat{c}}_{A(\lambda )}^{q}$) < 0, so if the residuals are negatively correlated with the variable its removal leads to an increase of cov (***s***, $\Phi_{A(\lambda )}{\hat{c}}_{A(\lambda )}^{q}$) as well.

Thus, as long as changes of the set $\Phi_{A(\lambda )}$ appear one at a time (which is ensured by the active set implementation), they do not influence the monotonous character of the estimate of the degrees of freedom.

More formally, when a variable *ϕ*_*j *_is added to the current set of variables $\Phi_{A(\lambda )}$, the solution for $\Phi_{A{\left(\lambda \right)}_{+}}=\Phi_{A(\lambda )}\cup \phi_{j}$ can be constructed from the solution of $\Phi_{A(\lambda )}$ in the following manner [39]:

(20) $$\Phi_{A{\left(\lambda \right)}_{+}}{\hat{c}}_{A{\left(\lambda \right)}_{+}}^{q}=\Phi_{A(\lambda )}{\hat{c}}_{A(\lambda )}^{q}+\hat{\gamma }u_{A{\left(\lambda \right)}_{+}}$$

where

(21) $$\hat{\gamma }=\min_{j\in A{\left(\lambda \right)}^{C}}^{+}\left\{\frac{\hat{D}-{\hat{d}}_{j}}{B_{A(\lambda )}-b_{j}}\right\}>0$$

is strictly positive by definition and gives the magnitude of the change.

(22) $$\hat{d}=\Phi^{T}(s-\Phi_{A(\lambda )}{\hat{c}}_{A(\lambda )}^{q})$$

is the vector of the current correlation and

(23) $$\hat{D}=\max_{j}\{{\hat{d}}_{j}|{\hat{d}}_{j}>0\}.$$

In addition,

(24) $$B_{A(\lambda )}={\left(1_{A(\lambda )}^{T}{\left(\Phi_{A(\lambda )}^{T}\Phi_{A(\lambda )}\right)}^{-1}1_{A(\lambda )}\right)}^{-\frac{1}{2}}$$

and

(25) $$u_{A(\lambda )}=\Phi_{A(\lambda )}B_{A(\lambda )}{\left(\Phi_{A(\lambda )}^{T}\Phi_{A(\lambda )}\right)}^{-1}1_{A(\lambda )}$$

leading to

(26) $$b=\Phi^{T}u_{A(\lambda )}.$$

We need to show that

(27) $$\begin{array}{l} \mathrm{cov}\left(s,\Phi_{A{\left(\lambda \right)}_{+}}{\hat{c}}_{A{\left(\lambda \right)}_{+}}^{q}\right)\geq \mathrm{cov}\left(s,\Phi_{A(\lambda )}{\hat{c}}_{A(\lambda )}^{q}\right)\\ \Leftrightarrow \mathrm{cov}\left(s,\Phi_{A{\left(\lambda \right)}_{+}}{\hat{c}}_{A{\left(\lambda \right)}_{+}}^{q}-\Phi_{A(\lambda )}{\hat{c}}_{A(\lambda )}^{q}\right)\geq 0\\ \Leftrightarrow s^{T}\left(\Phi_{A{\left(\lambda \right)}_{+}}{\hat{c}}_{A{\left(\lambda \right)}_{+}}^{q}-\Phi_{A(\lambda )}{\hat{c}}_{A(\lambda )}^{q}\right)\geq 0. \end{array}$$

Using the construction of $\Phi_{A{\left(\lambda \right)}_{+}}{\hat{c}}_{A{\left(\lambda \right)}_{+}}^{q}$ from above, this leads to

(28) $$\begin{array}{r} \Leftrightarrow s^{T}\left(\Phi_{A(\lambda )}{\hat{c}}_{A(\lambda )}^{q}+\hat{\gamma }u_{A{\left(\lambda \right)}_{+}}-\Phi_{A(\lambda )}{\hat{c}}_{A(\lambda )}^{q}\right)\geq 0\\ \Leftrightarrow s^{T}(\hat{\gamma }u_{A{\left(\lambda \right)}_{+}})\geq 0. \end{array}$$

It is known from its definition that $\hat{\gamma }$ is strictly positive, thus it can be dropped from the inequality and

(29) $$\begin{array}{r} s^{T}u_{+}\geq 0\\ \Leftrightarrow s^{T}\Phi_{A{\left(\lambda \right)}_{+}}B_{A{\left(\lambda \right)}_{+}}{\left(\Phi_{A{\left(\lambda \right)}_{+}}^{T}\Phi_{A{\left(\lambda \right)}_{+}}\right)}^{-1}1_{A{\left(\lambda \right)}_{+}}\geq 0. \end{array}$$

It is also known from the idea of the non-negative lasso that all variables in *X*_*A *_are positively correlated with the remaining residuals, so

(30) $$\begin{array}{l} \mathrm{cov}\left(\Phi_{A(\lambda )},s-\Phi_{A(\lambda )}{\hat{c}}_{A(\lambda )}^{q}\right)\geq 0\\ \Leftrightarrow {\left(s-\Phi_{A(\lambda )}{\hat{c}}_{A(\lambda )}^{q}\right)}^{T}\Phi_{A(\lambda )}\geq 0\\ \Leftrightarrow s^{T}\Phi_{A(\lambda )}\geq {\left(\Phi_{A(\lambda )}{\hat{c}}_{A(\lambda )}^{q}\right)}^{T}\Phi_{A(\lambda )}. \end{array}$$

Using this result,

(31) $$\begin{array}{l} s^{T}\Phi_{A{\left(\lambda \right)}_{+}}B_{A{\left(\lambda \right)}_{+}}{\left(\Phi_{A{\left(\lambda \right)}_{+}}^{T}\Phi_{A{\left(\lambda \right)}_{+}}\right)}^{-1}1_{A{\left(\lambda \right)}_{+}}\\ \geq {\left(\Phi_{A{\left(\lambda \right)}_{+}}{\hat{c}}_{A{\left(\lambda \right)}_{+}}^{q}\right)}^{T}\Phi_{A{\left(\lambda \right)}_{+}}B_{A{\left(\lambda \right)}_{+}}\\ {\left(\Phi_{A{\left(\lambda \right)}_{+}}^{T}\Phi_{A{\left(\lambda \right)}_{+}}\right)}^{-1}1_{A{\left(\lambda \right)}_{+}} \end{array}\;$$

holds true and it suffices to show that

(32) $$\begin{array}{l} {\left(\Phi_{A{\left(\lambda \right)}_{+}}{\hat{c}}_{A{\left(\lambda \right)}_{+}}^{q}\right)}^{T}\Phi_{A{\left(\lambda \right)}_{+}}B_{A{\left(\lambda \right)}_{+}}\\ {\left(\Phi_{A{\left(\lambda \right)}_{+}}^{T}\Phi_{A{\left(\lambda \right)}_{+}}\right)}^{-1}1_{A{\left(\lambda \right)}_{+}}\geq 0\\ \Leftrightarrow {\left(\Phi_{A{\left(\lambda \right)}_{+}}{\hat{c}}_{A{\left(\lambda \right)}_{+}}^{q}\right)}^{T}u_{A_{+}}\geq 0\\ \Leftrightarrow {\hat{c}}_{A{\left(\lambda \right)}_{+}}^{q}1^{T}\Phi_{A{\left(\lambda \right)}_{+}}^{T}u_{A{\left(\lambda \right)}_{+}}\geq 0. \end{array}$$

When further recalling the fact from [39] that $\Phi_{A(\lambda )}^{T}u_{A(\lambda )}=B_{A(\lambda )}1_{A(\lambda )}$, this can be reduced to

(33) $${\left({\hat{c}}_{A{\left(\lambda \right)}_{+}}^{q}\right)}^{T}B_{A{\left(\lambda \right)}_{+}}1_{A{\left(\lambda \right)}_{+}}\geq 0,$$

but as $B_{A{\left(\lambda \right)}_{+}}$ is strictly positive by definition, it follows that

(34) $$\begin{array}{r} {\left({\hat{c}}_{A{\left(\lambda \right)}_{+}}^{q}\right)}^{T}1_{A{\left(\lambda \right)}_{+}}\geq 0\\ \Leftrightarrow \sum_{i}{\left({\hat{c}}_{A{\left(\lambda \right)}_{+}}^{q}\right)}_{i}\geq 0. \end{array}$$

This is always fulfilled for the non-negative lasso as it is the constraint on its initial optimization problem. The case of the removal of *ϕ*_*k *_from $\Phi_{A(\lambda )}$ can be argued almost identically with the only difference being that now

(35) $$\Phi_{A{\left(\lambda \right)}_{+}}{\hat{c}}_{A{\left(\lambda \right)}_{+}}^{q}=\Phi_{A(\lambda )}{\hat{c}}_{A(\lambda )}^{q}+\tilde{\gamma }u_{A{\left(\lambda \right)}_{+}}$$

where

(36) $$\tilde{\gamma }=\underset{\gamma_{j}>0}{\min}\{\gamma_{j}\}$$

which is also always positive and thus can be dropped from the resulting inequality in exactly the same fashion as $\hat{\gamma }$ could be dropped for the case of the addition of a variable. Consequently, changes in $\Phi_{A(\lambda )}$ do not change the monotony of the GDF estimate.

### C Lower bound properties of BIC_*min*_

BIC_*min *_is a lower bound for BIC, if ∀*k *≥ *i*

(37) BIC_*min*_(*i*) ≤ BIC(*k*),

which equals

(38) $$\frac{N}{\sigma_{\varepsilon }^{2}}\text{MSE}+df(\lambda_{i})\log N\leq \frac{N}{\sigma_{\varepsilon }^{2}}\text{MSE}(\lambda_{i})+df(\lambda_{k})\log N$$

which is always fulfilled because MSE ≤ MSE(*λ*_*i*_) and *df *(*λ*_*i*_) ≤ *df *(*λ*_*k*_) for *i *≤ *k *and *N *≥ 1, $\sigma_{\varepsilon }^{2}$ > 0.

### D SNR definition for simulated spectra

Given the undistorted simulated signal **s**, the effect of Poisson noise is simulated with *s*_*i *_← *v*_*i*_, where *v*_*i *_is drawn from a Poisson distribution with mean *ks*_*i *_+ 1. The signal-to-noise ratio (SNR) thus depends on the parameter *k*. In order to determine *k *for a selected set of SNR values, we consider the definition

(39) $$\text{SNR}≐\frac{\sigma_{s}^{2}}{\sigma_{n}^{2}}.$$

The empirical variance of the original signal **s **multiplied by a scalar *k *is defined as

(40) $$\sigma_{s}^{2}(k)≐k^{2}\sum_{i=1}^{N}{\left(s_{i}-\bar{s}\right)}^{2},$$

where $\bar{s}$ denotes the mean over all *s*_*i*_. For Poisson noise, location and dispersion parameters coincide, i.e. with *X *~ P(*λ*) we have Var(*X*) = E(*X*) = *λ*, and we approximate the variance of a set of Poisson variables *n*_*i *_~ P(*ks*_*i*_), *i *= 1, ..., *N *by their average

(41) $$\sigma_{n}^{2}(k)≐\frac{1}{N}\sum_{i=1}^{N}ks_{i}.$$

For a given SNR, this allows the estimation of *k *because

(42) $$\text{SNR}=\frac{\sigma_{s}^{2}(k)}{\sigma_{n}^{2}(k)}=k\frac{\sigma_{s}^{2}}{\sigma_{n}^{2}}$$

and thus

(43) $$k=\frac{\sigma_{n}^{2}}{\sigma_{s}^{2}}\text{SNR}.$$

## Authors' contributions

BYR and MK have developed the methodology, implemented the software, carried out the data analysis and drafted the manuscript. HS and JAJS have contributed to the basic methodology and the manuscript, carried out critical review and provided application feedback and evaluation for the proposed methods. FAH has suggested the fractional averagine approach, and has contributed to the manuscript and the overall project design. All authors have read and approved the final manuscript.

## Supplementary Material

Additional file 1 **A zip folder containing all simulation files (R data files).** Click here for file

Additional file 2 **The zipped original LC/MS .wiff-file on which MarkerView was run (as acquired by the AB/Sciex QStar instrument)** Click here for file

Additional file 3 **The original spectrum of BSA-sample.wiff integrated over retention time (23.817-29.278 minutes) on which NITPICK was run.** Click here for file

Additional file 4 **A zip-folder containing all pepex results on the simulated data.** Click here for file

Additional file 5 **A zip-folder containing all NITPICK results on the simulated data sets and for each SNR (SNR in 5, 10, 25, 50, 100) a R data file called**
• resultList_0_’SNR’_0.1.RDA gives the peaks found by NITPICK for all spectra of a certain SNR
• lengthResultList_0_’SNR’_0.1.RDA gives the number of peaks found by NITPICK for each spectrum
• correct_0_’SNR’_0.1.RDA gives the number of correctly identified peaks found by NITPICK for each spectrum
• tooMany_0_’SNR’_0.1.RDA gives the number of incorrectly identified peaks found by NITPICK for each spectrum
• pp_resultList_0_3_0_’SNR’_0.1.RDA gives the peaks found by NITPICK for all spectra of a certain SNR after postprocessing with g=3
• lengthResultList_0_3_0_’SNR’_0.1.RDA gives the number of peaks found by NITPICK for each spectrum after postprocessing with g=3
• correct_0_3_0_’SNR’_0.1.RDA gives the number of correctly identified peaks found by NITPICK for each spectrum after postprocessing with g=3
• tooMany_0 3_0_’SNR’_0.1.RDA gives the number of incorrectly identified peaks found by NITPICK for each spectrum after postprocessing with g=3 Click here for file

Additional file 6 **Excel sheet containing the peaks detected by NITPICK (mz-position, charge, intensity) as well as their manual validation.** Click here for file

Additional file 7 **Excel sheet containing the peaks detected by MarkerView (mz-position, charge, if available) as well as their manual validation.** Click here for file
